# Roles of reactive oxygen species in inflammation and cancer

**DOI:** 10.1002/mco2.519

**Published:** 2024-04-04

**Authors:** Yunfei Yu, Shengzhuo Liu, Luchen Yang, Pan Song, Zhenghuan Liu, Xiaoyang Liu, Xin Yan, Qiang Dong

**Affiliations:** ^1^ Department of Urology West China Hospital Sichuan University Chengdu China

**Keywords:** cancer, immune, reactive oxygen species, treatment

## Abstract

Reactive oxygen species (ROS) constitute a spectrum of oxygenic metabolites crucial in modulating pathological organism functions. Disruptions in ROS equilibrium span various diseases, and current insights suggest a dual role for ROS in tumorigenesis and the immune response within cancer. This review rigorously examines ROS production and its role in normal cells, elucidating the subsequent regulatory network in inflammation and cancer. Comprehensive synthesis details the documented impacts of ROS on diverse immune cells. Exploring the intricate relationship between ROS and cancer immunity, we highlight its influence on existing immunotherapies, including immune checkpoint blockade, chimeric antigen receptors, and cancer vaccines. Additionally, we underscore the promising prospects of utilizing ROS and targeting ROS modulators as novel immunotherapeutic interventions for cancer. This review discusses the complex interplay between ROS, inflammation, and tumorigenesis, emphasizing the multifaceted functions of ROS in both physiological and pathological conditions. It also underscores the potential implications of ROS in cancer immunotherapy and suggests future research directions, including the development of targeted therapies and precision oncology approaches. In summary, this review emphasizes the significance of understanding ROS‐mediated mechanisms for advancing cancer therapy and developing personalized treatments.

## Graphical abstract

We reviewed the functions of ROS in normal cells, cancer cells and immune cells in the tumor microenvironment. Based on the unique roles of ROS in cancer immunity, ROS can affect current cancer immunotherapies and be the novel target for developing more immunotherapies.

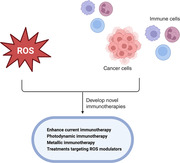

## 1 INTRODUCTION

Reactive oxygen species (ROS) epitomize an expansive array of oxidants originating from molecular oxygen. They are constituents of the broader category of reactive species, which encompasses reactive nitrogen, sulfur, carbon, selenium, electrophile, and halogen species. Capable of undergoing redox reactions (reduction–oxidation), they give rise to oxidative modifications on biological macromolecules, thereby participating in redox signaling and physiological processes.^1^ These species emerge from the one‐electron reduction of oxygen, including hydrogen peroxide (H_2_O_2_), superoxide anion (O_2_^−^), and hydroxyl radical (OH^−^).^2^ Mounting evidence underscores the pivotal role of ROS as critical intracellular and extracellular signaling entities, capable of both cellular harm and regulation, affecting lipids, proteins, and DNA. Crucial to the intricate balance of redox control are two primary enzyme systems, namely the glutathione (GSH) and thioredoxin (Trx) systems, governing the reductive pathways. These systems facilitate redox regulatory functions and the activities of antioxidant enzymes, utilizing reducing equivalents derived from NADPH.^3^, ^4^

Inflammation, a physiological response to infections and injuries, serves to restore homeostasis and safeguard tissue function.^5^ However, persistent local inflammation can promote cancer development and progression. Conditions like gastritis induced by *Helicobacter pylori* or chronic hepatitis are linked to the development of gastric and liver cancers, respectively.^6^ Many tumors exhibit a proinflammatory microenvironment where infiltrated immune cells release factors fostering the differentiation of normal cells and facilitating the survival and metastasis of cancer cells.^7^ Remarkably, ROS assumes a dual role in both inflammation and cancer. Physiologically, ROS at optimal concentrations are crucial for an effective immune response, while excessive ROS levels can lead to sustained inflammation, even progressing to sepsis.^8^ Elevated ROS levels, coupled with enhanced antioxidant capacity and the maintenance of redox homeostasis, play a pivotal role in promoting cancer progression and metastasis.^9^ Importantly, a high concentration of ROS can induce apoptosis in cancer cells.^10^

The unique roles of ROS in inflammation and cancer facilitate its impact on tumor immunotherapy. Extensive research has long underscored the significance of ROS within the realm of immunology, indicating the essential role of ROS in tumor immunotherapy. Notably, as early as 1946, scientists identified a correlation between H_2_O_2_ and the generation of antibodies in bacteria.^11^ Subsequently, during the 1970s, heightened attention was directed towards the existence and function of oxygen radicals in the context of immunity.^12^ Carp and Janoff^13^ demonstrated that polymorphonuclear leukocytes generated ROS, thereby diminishing serum elastase‐inhibitory capacity, thus exacerbating damage to neighboring connective tissue at sites of inflammation. In 1990, Weitzman and Gordon^14^ highlighted the potential critical role of ROS originating from inflammatory cells in the process of tumorigenesis, along with the prospect that antioxidants could prove effective in the chemoprevention of cancer. Subsequently, in the 2010s, the association of mitochondria with innate immune signaling was underscored, emphasizing the indispensable role of mitochondrial ROS in the process of bacterial eradication.^15^ In 2012, a new form of cell death termed Ferroptosis was proposed, which was dependent on intracellular iron and oxidative stress related to ROS, resulting in the nonapoptotic destruction of specific cancer cells.^16^ More recently, the critical role of ferroptosis in cancer immunity has come to light. Wang et al.^17^ in 2019 elucidated how CD8+ T cells could downregulate the glutamate–cystine antiporter system xc‐, fostering the accumulation of lipid peroxidation and prompting ferroptosis in cancer cells. In 2022, Kim et al.^18^ elucidated how the collaboration of ferroptosis with myeloid‐derived suppressor cells (MDSCs) facilitated tumor progression by instigating immune suppression within the context of cancer.

This review comprehensively examines the multifaceted role of ROS in both immunological processes and tumorigenesis. It covers ROS sources, signaling pathways, and their impacts on normal cells. The discussion extends to ROS roles in inflammation, inflammation‐induced tumorigenesis, and cancer. Emphasizing the significance of ROS in cancer immunity, the focus of this review delves into the multifaceted implications of ROS in the intricate balance of immune responses and the progression of cancer. By highlighting various ROS‐mediated pathways, the review aims to offer insights into potential therapeutic targets and innovative approaches for cancer treatment and immunotherapy. It bridges recent advancements with a comprehensive overview of ROS in both physiological and pathological contexts, contributing to a clearer understanding and paving the way for future research and interventions.

## 2 ROS GENERATION AND SIGNALING PATHWAYS

**FIGURE 1:**
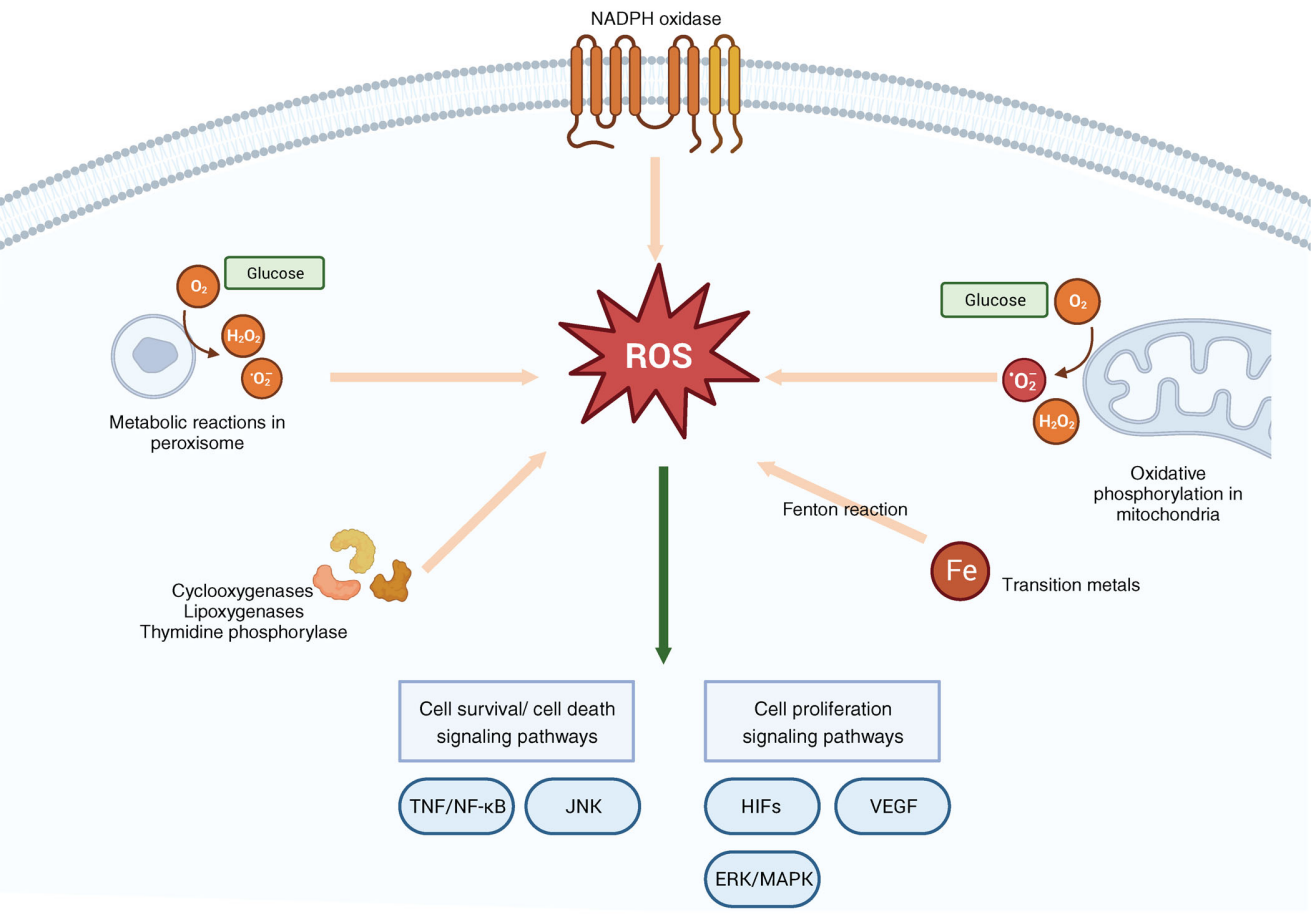
Generation and signaling pathways of ROS. ROS emanates from diverse sources, notably oxidative phosphorylation transpiring in the electron transport chain on the inner mitochondrial membrane. Concurrently, metabolic reactions within peroxisomes contribute to ROS production. Another subset of ROS is engendered by nicotinamide adenine dinucleotide phosphate (NADPH) oxidases (NOX), which catalyze the conversion of oxygen into superoxide. Additionally, ROS can stem from cyclooxygenases, lipoxygenases, and thymidine phosphorylase. Significantly, transition metals, particularly iron, can initiate ROS formation independently of enzymatic processes through a Fenton reaction. Noteworthy is the involvement of ROS in cell metabolism signaling pathways, influencing the survival, death, and proliferation of cells. TNF, tumor necrosis factor; JNK, c‐jun N‐terminal kinase; HIFs, hypoxia‐inducible transcription factors; VEGF, vascular endothelial growth factor; ERK/MAPK, the extracellular‐signal‐regulated kinase/mitogen‐activated protein kinase pathway.

Mitochondria and peroxisome are the primary sources of endogenous ROS, engendering intracellular oxidative stress.^19^ The generation of ROS primarily occurs within the electron transport chain (ETC) situated on the inner mitochondrial membrane. This process, integral to oxidative phosphorylation, facilitates the generation of adenosine triphosphate (ATP) from oxygen and simple sugars.^20^ Notably, this intricate process involves five pivotal protein complexes, namely NADH (complex I), succinate (complex II), ubiquinol (complex III), cytochrome *c* oxidase (complex IV), and F1F0‐ATP synthase (complex V).^21^ The electron leakage at these complexes promotes the production of superoxide. Subsequently, superoxide is converted to H_2_O_2_ by superoxide dismutase 1 (SOD1) in the intermembrane space.^20^ These molecular processes culminate in the generation of superoxide and H_2_O_2_, the principal ROS emanating from mitochondria. Additionally, a subset of ROS is produced by nicotinamide adenine dinucleotide phosphate (NADPH) oxidases (NOX), a transmembrane protein family orchestrating the transport of electrons across biological membranes, catalyzing the conversion of oxygen into superoxide. Among these, the catalytic subunit, NADPH oxidase 2 (NOX2), manifests a prominent role.^22^ The NOX family comprises six distinct NOX2 variants discernible in diverse tissue contexts.^23^ Furthermore, ROS can also derive from cyclooxygenases, lipoxygenases, and thymidine phosphorylase.^24^ Transition metals, notably iron, can engender ROS independently of enzymes through the Fenton reaction.^25^ Notably, Fe^2+^ can react with H_2_O_2_ to yield a hydroxy radical, causing damage to DNA and other related molecules^26^ (Figure 1).

The cellular generation of ROS and reactive nitrogen species (RNS) during metabolic processes Is inevitable, often characterized as detrimental to cellular integrity. However, under specific conditions, these species assume pivotal biological roles within cellular signaling pathways.^27^ ROS orchestrates crucial regulatory functions in the modulation of tumor necrosis factor (TNF) and TNF receptor signaling, exerting influence over cell survival or cell death.^28^ Pertinently, targeted inhibition of mitochondrial ROS in monocytes and T cells via the mitochondria‐specific antioxidant MitoVit E has validated the significance of mitochondrial ROS in the activation of the NF‐κB signaling pathway, a downstream mediator of TNF signaling.^29^ Furthermore, ROS serve as triggering agents for key components of the c‐Jun N‐terminal kinase (JNK) and p38 kinase (p38MAPK) pathways, thereby initiating the apoptotic cascade.^30^ Activated JNK translocates to the mitochondrial membrane, intensifying ROS production, subsequently perpetuating JNK activation through a positive feedback loop mediated by mitochondrial ROS.^31^ The inhibition of nitric oxide (NO) synthesis has been demonstrated to impede insulin‐stimulated glucose transport and glucose transporter 4 recruitment, underscoring the positive regulatory role of NO in insulin signaling.^32^

The endogenous ROS can interact with proteins, regulating different signaling pathways of cell metabolism. ROS can activate nonreceptor protein kinases of the Src family, small G proteins, and the tyrosine kinase receptors of growth factor.^33^, ^34^ The essential role of mitochondrial ROS in stabilizing hypoxia‐inducible transcription factors (HIFs) under hypoxic conditions has been a subject of research for several decades.^35^, ^36^ Stabilized HIFs prompt the initiation of a comprehensive transcriptional program, encompassing genes vital to angiogenesis.^37^ The angiogenic process necessitates the proliferation of endothelial cells,^38^ which is facilitated by the upregulation of vascular endothelial growth factor (VEGF) expression mediated by HIF. VEGF binding to its receptor activates crucial signaling pathways pivotal to endothelial cell proliferation.^39^ Moreover, activation of the extracellular‐signal‐regulated kinase/mitogen‐activated protein kinase (ERK/MAPK) pathway can amplify the mitogenic effects of VEGF, thus potently stimulating cellular proliferation.^40^

## 3 THE IMPACTS OF ROS ON NORMAL CELLS

Redox biology significantly influences numerous physiological processes in normal cellular function, including the facilitation of extracellular matrix formation, regulation of wound healing, and modulation of immune responses.^41^, ^42^, ^43^ Extensive evidence underscores the enduring impact of ROS on cellular function through the modification of epigenetic biomolecules. Conversely, in the short term, ROS is implicated in three distinctive forms of cell death, namely apoptosis, necrosis, and autophagy. Furthermore, ROS can intricately interact with transcription factors (TFs), thereby exerting additional regulatory effects on cellular metabolism.

### 3.1 Long‐term impacts of ROS on normal cells

The redox state exerts a persistent influence on cells by intricately modulating epigenetic processes, such as the methylation of DNA and histone methylation.^44^ Under conditions of oxidative stress, the upregulation of GSH synthesis serves as a crucial antioxidant defense mechanism, while enzymes responsible for regenerating methionine from homocysteine experience downregulation.^45^ Notably, S‐adenosyl methionine, derived from methionine, serves as the methyl donor for DNA‐methyltransferases. Furthermore, select metabolites from the mitochondrial tricarboxylic acid (TCA) cycle can serve as substrates and regulators for epigenetic modifier enzymes. Histone acetylases catalyze histone acetylation, utilizing acetyl‐CoA as a substrate donor.^46^ ROS can also influence energy metabolism in cells. In the mitochondrial TCA cycle, cysteine residues in isocitrate dehydrogenase and a‐KGDH can turn into an inactivated state under higher H_2_O_2_ conditions, limiting the function of NADH for the ETCs.^47^ Additionally, under H_2_O_2_ treatment, cys392, a cysteine residue, undergoes S‐CoAlation (protein thiol modification by coenzyme A) in mice, leading to the deactivation of pyruvate dehydrogenase kinase 2 and the inhibition of pyruvate dehydrogenase phosphorylation.^48^ The increased expression of catalase in astrocytes modulates various biological pathways in neurons, including carbohydrate, lipid, and amino acid metabolism. Furthermore, ROS in astrocytes exhibit a neuroprotective function by activating nuclear factor erythroid 2‐related factor 2 (NRF2), subsequently inhibiting NOX enzymes and reducing extracellular ROS levels.^49^ Redox biology significantly influences proteostasis, encompassing crucial processes such as protein biosynthesis, folding, quality control, and degradation. Redox‐dependent regulation impacts mRNA translation, including initiation, elongation, and termination processes.^50^, ^51^ Phosphorylation of eIF2, a translational initiation factor, is regulated by kinases sensitive to the redox state, ultimately leading to global repression of mRNA translation.^52^

### 3.2 The short‐term impacts of ROS on cells by causing cell death

Oxidative stress constitutes a potent catalyst for three classic types of cell death, encompassing apoptosis, necrosis, and autophagy.^53^ In eukaryotes, mitochondria‐derived ROS are implicated in the induction of cell death. A point mutation observed in cerevisiae cells has been linked to the highly conserved AAA ATPase Cdc48/VCP, disrupting the connection between Cdc48 and Vmsl1. This dissociation results in the aberrant localization of Cdc48 to mitochondria, facilitating the production of elevated levels of ROS from impaired mitochondria, ultimately leading to apoptosis.^54^, ^55^ In another aspect, prodeath proteins BAX and BAK contribute to mitochondrial outer membrane permeabilization (MOMP), prompting the release of mitochondrial prodeath molecules, including the hemoprotein cytochrome *c*, subsequently promoting the formation of the apoptosome and activating caspase‐3 and caspase‐7. Remarkably, it was found that in the process of apoptosis, cardiolipin was oxidized by the peroxidase activity of the cytochrome *c* hemoprotein, which is crucial for subsequent cytochrome *c* release and the execution of apoptosis.^56^ Autophagy serves as a critical mechanism for maintaining cellular homeostasis under conditions of oxidative stress, facilitating the degradation of damaged cellular components and the recycling of cellular contents. p53 has been implicated in oxidative stress‐related autophagy processes, as evidenced by delayed tissue damage and extended lifespan observed in mice following p53 and Atg7 gene knockdown.^57^ Oxidative stress can trigger necrosis, wherein NF‐kB serves as a vital modulator, suppressing the Nrf2–ARE antioxidant pathway via transcriptional silencing and potentially inhibiting the expression of prosurvival genes, such as Nrf2.^58^ Recent advancements have led to the recognition of a novel form of oxidative and iron‐dependent cell death termed ferroptosis, with system x_c_^−^–GSH–GPX4 pathway playing an essential role in its initiation. Notably, phospholipid hydroperoxides derived from lipids, along with their precursors, polyunsaturated fatty acids, have been found to intimately associate with the mechanistic pathways of ferroptosis.^59^ Moreover, p53 participates in the regulation of ferroptosis in conjunction with other inducers, such as GPX4 inhibitors or heightened levels of ROS.^60^ However, p53 exhibits a complex role in attenuating cell death, as it can induce ROS production leading to apoptosis and ferroptosis,^61^, ^62^ while also demonstrating antioxidant activity to mitigate cellular damage accumulation.^63^

### 3.3 The impacts of ROS on TFs

Redox regulation is also connected with transcription factors (TFs). Further, TFs can interact with corresponding genes to regulate redox homeostasis (Table 1). NRF2, acknowledged as a stress‐responsive TF, adeptly discerns alterations in cellular redox status through its cysteine‐rich adaptor kelch‐like ECH‐associated protein 1 (KEAP1). Elevated ROS levels induce conformational changes through the oxidation of cysteines in KEAP1, liberating NRF2 and facilitating its translocation to the nucleus. Subsequently, NRF2 targets enzymes crucial for redox homeostasis and cofactor generation pivotal to redox reactions.^64^ Hypoxia‐inducible factor 1α (HIF1α) can promote energy production through glycolysis under low oxygen levels, maintaining redox status.^65^ Under normal oxygen levels, HIF1α undergoes hydroxylation by prolyl hydroxylase PHD2, leading to its degradation. Contrastingly, oxidative stress can deactivate PHD2 through oxidative dimerization, culminating in the activation of HIF‐1α and the induction of aerobic glycolysis in response to stress.^66^ The forkhead box O (FOXO) family of TFs also contributes to the maintenance of redox balance. MnSOD, a FOXO‐regulated antioxidant, catalyzes the dismutation of oxygen reduction products to produce oxygen and H_2_O_2_.^67^ Catalase, a peroxisomal heme peroxidase regulated by FOXO3a, facilitates the dismutation of H_2_O_2_ to water and oxygen.^68^ Alterations in ROS levels can prompt posttranslational modifications of FOXO, including phosphorylation, acetylation, and ubiquitination, ultimately governing the subcellular localization, activity, and stability of FOXO.^69^ The BTB and CNC homology 1 (BACH1), a heme‐binding TF and an inhibitor of heme oxygenase 1 (HMOX1), contributes significantly to the oxidative stress response. HMOX1 facilitates the conversion of heme to ferrous iron and biliverdin, further progressing to bilirubin, an effective radical scavenger.^70^ A study has reported that BACH1 knockdown influences genes implicated in heme degradation (HMOX1, FTL, SCL48A1) and redox regulation (GCLC, SCL7A11), underscoring the involvement of BACH1 in the response to oxidative stress.^71^ Additionally, Wiel et al.^72^ established that BACH1 promotes the transcription of hexokinase 2 and GAPDH, thereby upregulating glucose uptake, glycolysis rates, and lactate secretion, ultimately fostering glycolysis‐dependent metastasis in lung cancer.

**TABLE 1:** Major TFS and their related genes in the regulation of redox homeostasis.

TFs	Related genes that interact to regulate redox homeostasis	References
NRF2	Keap1, STAT3, HO‐1, TIGAR, AMPK, KRAS, NRG1, PRAK, p53	^73^, ^74^, ^75^, ^76^, ^77^, ^78^, ^79^, ^80^, ^81^
HIF‐1a	PHD, FIH‐1, COX, LON, NDUFA4L2, ELAVL1, HO‐1	^82^, ^83^, ^84^, ^85^, ^86^, ^87^
FOXO	AKT, JNK, SIRT1, AMPK, transporting 1	^88^, ^89^, ^90^, ^91^, ^92^
BACH1	NRF2, HO‐1, AKT, mTOR, c‐Myc	^93^, ^94^, ^95^, ^96^

Abbreviations: AKT: protein kinase B; AMPK: AMP‐activated kinase; BACH1: The BTB and CNC homology 1; COX: cytochrome *c* oxidase; FOXO: forkhead box O; HO‐1: heme oxygenase‐1 signaling; HIF‐1a: hypoxia‐inducible transcription factors; JNK: c‐jun N‐terminal kinase; Keap1: kelch‐like ECH‐associated protein 1; mTOR: mammalian target of rapamycin; NRG1: neuregulin 1; NRF2: nuclear factor erythroid 2‐related factor 2; PRAK: p38‐regulated/activated protein kinase; PHD: prolyl hydroxylase domain protein; STAT3: signal transducer and activator of transcription 3; SIRT1: sirtuin 1; TFs: transcription factors; TIGAR: TP53‐induced glycolysis and apoptosis regulator.

## 4 THE CROSSTALK BETWEEN ROS AND INFLAMMATION

Considerable research endeavors have delved into elucidating the intricate interplay between ROS and inflammation. Oxidative stress induced by ROS manifests a bidirectional interaction with inflammation, culminating in a feedback loop. Inflammatory cells and related cytokines can result in redox imbalances. Evidence of DNA oxidative damage has been documented in hepatocytes and lung epithelial cells cocultured with activated neutrophils.^97^ Melatonin, through the ROS/TXNIP/HIF‐1α axis, exhibits the capacity to modulate T cells, thereby alleviating autoimmune uveitis.^98^ Inflammatory cytokines serve to augment ROS accumulation in phagocytic and nonphagocytic cells, instigating oxidative stress in both acute and chronic pathological conditions.^99^, ^100^ TNF‐α has been demonstrated to impede oxidative phosphorylation in a hippocampal cell line, underscoring the detrimental impact of inflammation on mitochondrial function.^101^ Furthermore, in instances of sepsis and acute inflammation, the balance of redox homeostasis is disrupted, characterized by a prooxidant state and excessive production of ROS and RNS, ultimately leading to endothelial cell damage.^102^

Besides, cellular redox status regulates the inflammation response. During the formation of inflammation, the functions of immune cells can be altered, turning into a phenotype with increased production of proinflammatory cytokines and ROS, fostering the progression of chronic inflammation and genome oxidative damage.^103^ Elevated ROS production and subsequent oxidative stress can incite inflammatory responses and apoptosis, ultimately resulting in hepatic steatosis and liver tissue injury.^104^ Yu et al.^105^ have demonstrated that amplified mitochondrial mass in macrophages precipitates excessive ROS generation, subsequently activating the NF‐κB pathway and steering the proinflammatory differentiation of macrophages. The escalation of ROS levels from inflammatory cells can intensify inflammation, while ROS‐dependent inflammation engenders further oxidative stress.^106^ Gong et al.^107^ suggest that the administration of sulforaphane, an antioxidant, in a mouse model, can counteract cell death and suppress the activation of microglia and the inflammasome, underscoring the pivotal role of ROS in the upstream pathways of inflammation. Additionally, mitochondrial dysfunction can trigger an upsurge in inflammatory signaling through the cyclic GMP–AMP synthase (cGAS)‐stimulator of interferon genes (STING) pathway.^108^ Treatment with chebulinic acid in mice has been found to mitigate oxidative stress, thereby alleviating lipopolysaccharide (LPS)‐induced inflammation.^109^

ROS is intricately linked with factors contributing to the inflammatory response, including HIF‐1α, Wnt/β‐catenin, NF‐κB, and growth factors.^110^ In the progress of inflammation, immune cells release inflammatory biomolecules, enhancing vascular permeability and facilitating the migration of leukocytes to the injury site.^111^ Simultaneously, ROS plays a pivotal role in regulating the expression of pertinent proteins like intercellular adhesion molecule 1, vascular cell adhesion molecule 1, and selectin in endothelial cells, which can interact with leukocytes and promote their migration.^112^ Inflammatory cytokines such as TNF, platelet‐derived growth factor, angiopoietin‐1, and VEGF induce immune cell migration and adhesion by binding to corresponding receptors on the cell surface, activating NADPH oxidases to generate ROS.^113^, ^114^

## 5 THE ROLES OF ROS IN INFLAMMATION‐INDUCED TUMORIGENESIS

### 5.1 Inflammation and ROS are crucial in tumorigenesis

The immune system plays a crucial role in tumorigenesis. Studies have demonstrated the substantial presence of inflammatory cells, including T cells, macrophages, and neutrophils, within various types of solid tumors, highlighting their significant involvement in the tumor microenvironment (TME).^115^ Notably, CD8^+^ T cells serve as direct executioners, integral to the landscape of cancer immunotherapy.^116^ CD4^+^ helper T cells (Th), including Th1 and Th2, can secret inflammatory cytokines that exert influence over the proliferation and cytotoxicity of CD8+ T cells.^117^ FasL and PDL‐1 in TME can lead to the apoptosis and exhaustion of CD8^+^ T cells, resulting in tumor immune escape.^118^ The accumulation of neutrophils in the TME is linked to the initiation and progression of tumors, often serving as a marker of unfavorable clinical outcomes across several cancer types.^119^ Tumor‐associated macrophages (TAMs) has been found to be involved in regulating tumorigenesis, tumor invasion and chemoresistance.^120^ TAMs foster tumor cell growth and angiogenesis by secreting associated growth factors such as transforming growth factor‐β, epidermal growth factor (EGF), and VEGF.^121^

Moreover, immune cells and inflammatory cytokines frequently engender a state of redox imbalance in response to infections, trauma, and cancer. Activated neutrophils and macrophages produce substantial quantities of ROS to counteract pathogens. Proinflammatory cytokines contribute to the accumulation of ROS within cells, thereby inducing oxidative stress observed in various chronic diseases. Notably, TNF‐α has been associated with the proinflammatory differentiation of macrophages and the excessive generation of ROS.^99^, ^100^ The presence of accumulated ROS has been implicated in the promotion of intrahepatic cholangiocarcinoma initiation via JNK‐mediated cholangiocyte proliferation and oncogenic transformation triggered by TNF.^122^ In gastritis, inflammation induces the activation of the NOX1 complex and the subsequent elevation of ROS levels through the TNF‐α/NF‐κB pathway. The NOX1/ROS signaling cascade is purported to play a crucial role in promoting abnormal proliferation of gastric epithelial cells within the inflamed mucosa, fostering gastric tumorigenesis.^123^ Additionally, ROS has the capacity to disrupt DNA repair pathways. Lower base excision repair efficiency has been observed in cancer patients relative to healthy individuals, primarily attributed to the inhibition of DNA repair enzymes by ROS. Furthermore, cytokine‐induced NO production has been shown to suppress specific DNA repair proteins.^124^, ^125^

### 5.2 ROS promote DNA damage and genomic instability induced by inflammation

ROS can interact with chronic inflammation in different steps of tumorigenesis, including cell malignant transformation, proliferation, migration. ROS has been recognized as a crucial molecule driving DNA damage and genomic instability induced by inflammation during carcinogenesis.^126^ The relationship between inflammation and genomic instability has been stated in multiple studies. Coculturing normal cells with activated macrophages and neutrophils has been shown to increase the incidence of genetic damage, including DNA strand breaks and sister chromatid exchanges.^127^, ^128^ Neutrophils can activate certain carcinogens, such as benzopyrene, aflatoxins, and quartz particles, thereby exacerbating genomic instability.^129^, ^130^ Additionally, 8‐nitroguanine, a byproduct of nitrative DNA damage, has been found to be notably concentrated in patients infected with tumor‐associated viruses, including human papillomavirus, helicobacter pylori, and Epstein–Barr virus.^131^, ^132^ It is pertinent to note that DNA oxidative damage and mutations frequently manifest at sites of tumorigenesis induced by infections and chronic inflammation.^133^

Extensive research has illuminated the association between inflammation‐induced DNA damage and mutations, primarily linked to the intricate interplay of ROS and RNS.^134^ The accumulation of hepatic neutrophils has been shown to regulate ROS production and elicit telomeric damage, a process that can be effectively reversed through the application of antioxidants.^127^ Furthermore, the infiltration of inflammatory cells in adjacent epithelial cells has been found to stimulate the generation of ROS, consequently fostering DNA damage.^135^ In the context of psoriasis, the heightened concentration of phagocytes has been implicated in ROS production, engendering DNA mutations and facilitating the perpetuation of these mutated cells, thereby promoting the onset of skin cancer.^136^ A study suggested that SIRT3 deficiency, a mitochondrial deacetylase, can elevate mitochondrial ROS levels, rendering cells more vulnerable to transformation and fostering tumor development.^137^

## 6 THE ROLES OF ROS IN CANCER

### 6.1 The dual roles of ROS in cancer

ROS wield a dual role in the context of cancer, representing both a contributing factor and a potential suppressor of malignancy. Elevated ROS levels have been observed across various cancer types, concomitant with oxidative DNA damage and the onset of genomic instability. The upregulation of antioxidant and detoxifying systems enables cancer cells to survive under heightened oxidative stress.^138^ The enhanced ROS levels within cancer cells sustain the activation of protumorigenic signaling pathways associated with evading cell death, facilitating angiogenesis, and promoting invasion and metastasis.^139^ Notably, the glycolytic shifts occurring in cancer cells contribute to ROS production, thereby sustaining malignancy and influencing cancer stem cells, which serve as a pivotal driver of tumor recurrence and therapeutic resistance.^140^ Conversely, certain studies have demonstrated the antitumor functions of ROS. In a mouse model of lung cancer, the administration of antioxidants was shown to accelerate tumor progression and reduce overall mouse survival.^141^ Inhibiting the antioxidant systems of cancer cells can lead to the suppression of tumor growth in mouse burdened with breast cancer or colon cancer.^142^ It has been suggested that ROS can impede tumor progression, partly by activating antitumor pathways and inducing the senescence or apoptosis of cancer cells.^143^

### 6.2 NRF2 is crucial for supporting the roles of ROS in cancer

**FIGURE 2:**
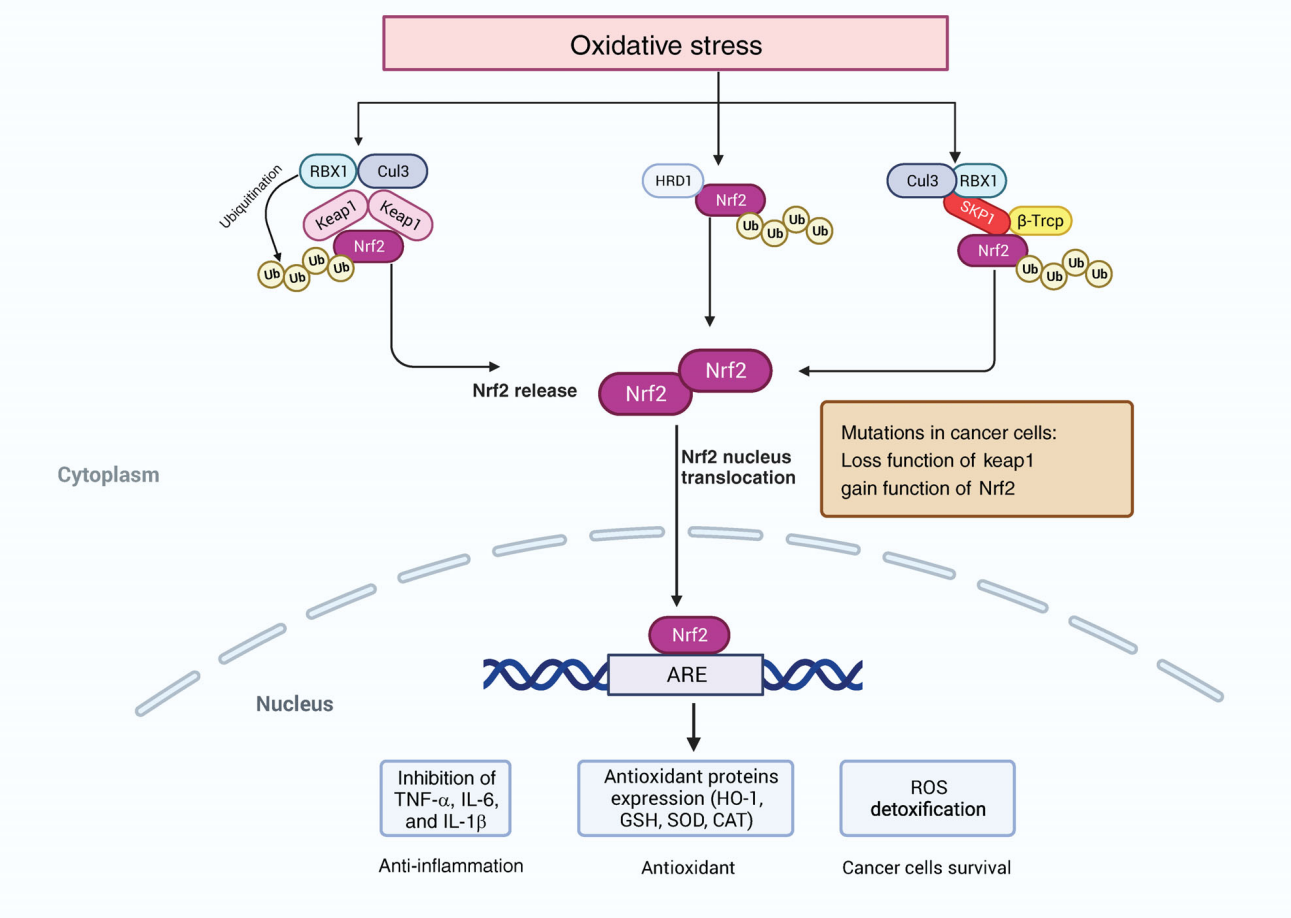
Activation of NRF2 pathway under oxidative stress. The regulation of NRF2 involves three distinct E3 ubiquitin ligase complexes: the KEAP1–CUL3–RBX1 complex, the β‐TrCP–SKP1–CUL1–RBX1 complex, and HRD1. When exposed to oxidative stress, ubiquitination processes transpire within these complexes, leading to the liberation of Nrf2. Upon its migration to the nucleus, Nrf2 binds to the antioxidant response element (ARE), triggering the expression of antioxidant proteins. This activation suppresses the release of proinflammatory cytokines and facilitates the detoxification of ROS. Nrf2, nuclear factor erythroid 2‐related factor 2; Keap, kelch‐like ECH‐associated protein 1; Cul3, Cullin‐3; RBX1, Ring box 1; β‐TrCP, β‐ transducin repeats‐containing proteins; SKP1, the S‐phase Kinase‐Associated Protein 1; HRD1, HMG‐coA reductase degradation protein 1; HO‐1, heme oxygenase‐1 signaling; GSH, glutathione; SOD, superoxide dismutase 1; CAT, catalase.

Among those modulators, the TF NRF2 plays a vital role in cancer cells and the cellular antioxidant response. Many studies have demonstrated that activation of NRF2 in cancer cells can promote tumor progression and metastasis.^144^, ^145^, ^146^ NRF2 is involved in redox homeostasis through multiple mechanisms. It can regulate GSH metabolism, including xCT, GCLC/GCLM, and GS. The expression of the antioxidant enzyme system (GPX, GR, and PRX) and their cofactors, such as NADPH and FADH2, are also regulated by NRF2 to alter redox homeostasis.^147^, ^148^ In normal cells, NRF2 is regulated by three kinds of E3 ubiquitin ligase complexes, including the KEAP1–CUL3–RBX1 complex, the β‐TrCP–SKP1–CUL1–RBX1 complex and HRD1.^149^ The expression of NRF2 is found in all cell types and is limited in the cytoplasm, targeted for proteasomal degradation via KEAP1 to maintain its low level of basal protein. Enhanced ROS can induce the activation of NRF2, which in turn regulates cellular redox state by inducing enzymes that inhibit the accumulation of intracellular ROS.^150^ Increased oxidative stress induces alkylation or oxidation of KEAP1 cysteines, a formational change of KEAP1 that causes the liberation of NRF2 from KEAP1–E3 complex, suppression of ubiquitylation, and degradation of NRF2, promoting translocation of NRF2 to the nucleus to activate antioxidant genes.^151^ The role of NRF2 in cancer is complex. At the early stage of tumorigenesis, loss of NRF2 promotes epithelial–mesenchymal transition by increased ROS to support migration and invasion. Abnormal activation of NRF2 and its accumulation is associated with tumor‐promoting effects in cancer. Mutations in NRF2‐activation and KEAP1 preventing NRF2 repression occur in multiple types of cancer, such as liver cancer and lung cancer, leading to persistent activation of NRF2 in cancer cells.^152^, ^153^ Consistently, promoter methylation resulting in KEAP1 inactivation, p62‐mediated sequestration, and regulations of the oncometabolite fumarate and methylglyoxal can also cause NRF2 accumulation.^154^, ^155^, ^156^ NRF2 regulates several metabolic pathways of antioxidant defense and proliferative processes, including the pentose phosphate pathway (PPP) and serine synthesis pathway.^157^, ^158^ The function of NRF2 to ROS detoxification helps cancer cells survive under an oxidative environment (Figure 2).

### 6.3 The roles of ROS modulators in cancer

Other ROS modulators are also nonnegligible in supporting tumor progression. GSH is found to prevent DNA damage and maintain protein stability in cancers. The GSH‐S‐transferases (GST) and GSH peroxidases (GPXs) enzymes involved in the GSH pathway also contribute to tumor progression.^159^, ^160^ GST can promote the expression of oncogenic proteins, such as Akt, while GPXs are essential to reach a balance when ROS generation in tumor progression.^161^, ^162^ The thioredoxin (Trx) system supports tumor growth, including Trx and Trx reductase 1 (TrxRD1).^163^ The Trx system is associated with the regeneration of PRDX. The expression of PRDX1 and PRDX4 increases in cancer cells and promotes tumor progression.^164^, ^165^ Furthermore, inhibiting SOD1 leads to the suppression of lung tumorigenesis.^166^ In non‐small‐cell lung cancer, targeting SOD1 or TrxRD can cause enhanced exposure to ROS, which is harmful to cancer cell survival.^167^ The generation and reduction of NADPH are crucial for tumor survival. Glucose‐6‐phosphate dehydrogenase (G6PD) is a key enzyme for the generation of NADPH.^168^ It is found that G6PD‐deficient patients with lower NADPH levels are less susceptible to colorectal cancer.^169^

Supplementation of exogenous antioxidants, mostly N‐acetyl cysteine (NAC) and vitamin E, is utilized as a therapy for interfering with tumor processes. Application of NAC to p53 loss mice can inhibit DNA oxidation and genic mutation, impairing lymphoma and lung cancer survival.^170^ NAC can also affect the stabilization of HIF1a and disturb tumor growth of hepatocellular xenografts.^171^ However, other studies indicated that NAC treatment supported the initiation, progression and metastasis of several mouse models of melanoma and lung cancer.^141^, ^172^ Vitamin E is also recognized as an antitumor supplement. A multicenter clinical trial found that vitamin E supplementation group has a higher incidence of prostate cancer and this trial was stopped quickly.^173^ Glutamate‐cysteine ligase (GCL) is a rate‐limiting enzyme in the GSH metabolism. Anderton et al.^174^ suggested that inhibition of GCL by the MYC‐induced microRNA miR‐18a promoted the depletion of GSH in liver cancer, followed by increased sensitivity to oxidative stress in tumors. Buthionine‐(S, R)‐sulfoximine (BSO) is a drug inhibiting GCL activity for cancer therapy, further suppressing GSH synthesis and leading to GSH depletion.^175^, ^176^ However, an early clinical study demonstrated that BSO had limited clinical benefits as an anticancer treatment.^177^ Research from Nishizawa et al.^178^ indicated that tumors with low levels of GSH appeared to be more sensitive to the BSO treatment, and inhibiting GCL resulted in ferroptosis in cancer cells.

## 7 THE IMPACT OF ROS ON CANCER IMMUNITY IN THE TME

The TME is made up of different cell types, including tumor cells, noncancer stroma cells, tumor‐infiltrating leukocytes, and microbial populations. With those neighbor cells and surrounding molecules, cancer cells develop specific mechanisms to survive even in the conditions of hypoxia, higher ROS level, and lower pH.^179^, ^180^ Notably, ROS plays a dual role in the immune response of the TME. Activated T cells and NK cells can produce ROS, then promote the recruitment of neutrophils and macrophages to kill cancer cells.^181^ While the increased level of ROS can promote the survival of cancer cells by regulating tumor‐contributing immune cells, such as MDSCs, regulatory T cells (Tregs), and TAMs. Furthermore, ROS in TME produced by other cells can also contribute to the inadequate response of T cells in cancer patients.^182^ It was also found that oxidative stress could induce the apoptosis of Treg cells, and the apoptotic Treg cells contributed to the immunosuppressive environment in the TME, which was an emerging mechanism of tumor‐immune evasion.^183^ In the following section, we provide an insight to how ROS affects immune cells in the TME.

### 7.1 T cells

**FIGURE 3:**
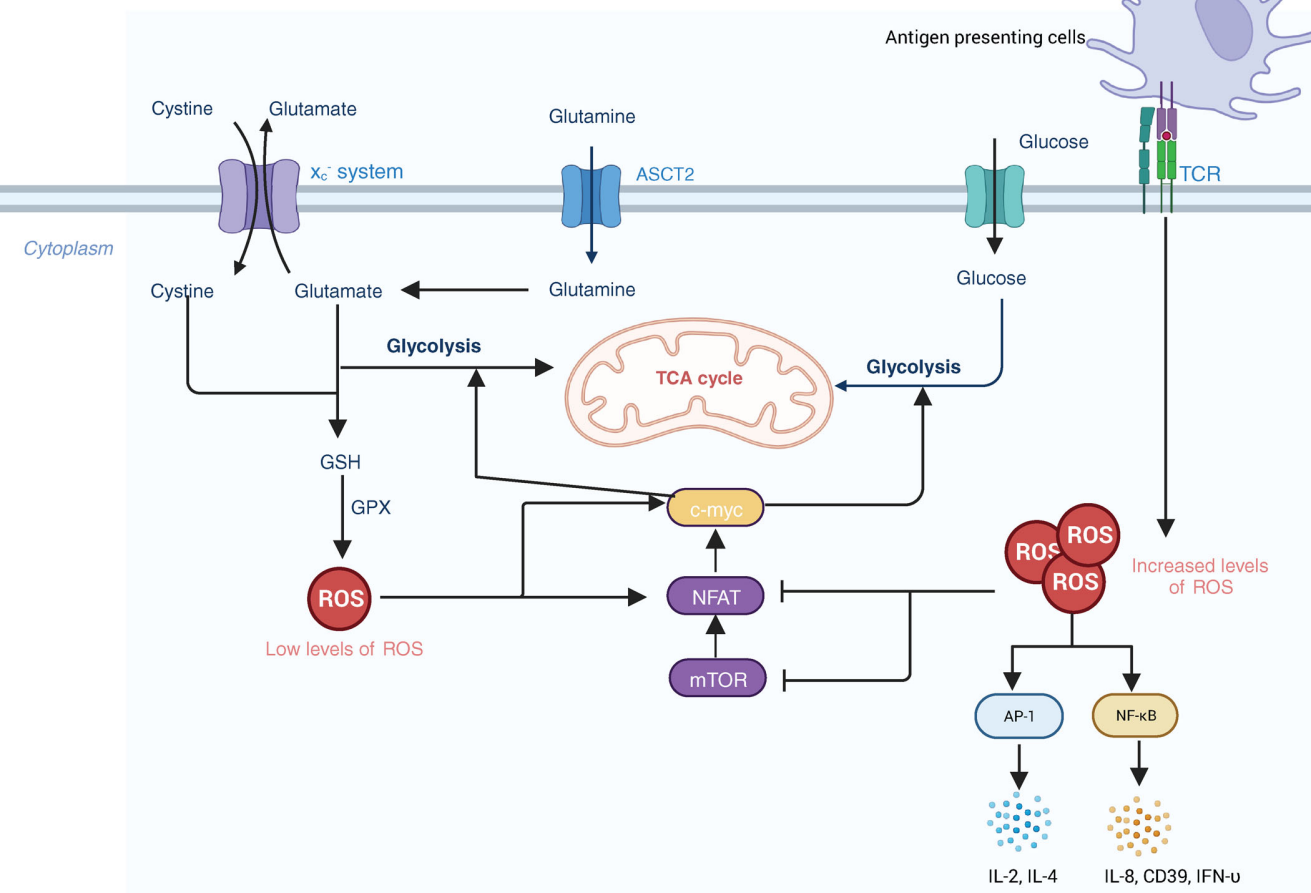
ROS involves in the process of metabolism of activated T cell. T cells are activated when the MHC of antigen presenting cells bind to the TCR. In activated T cells, ROS at low levels stimulate the expression of NFAT and c‐myc, thereby accelerating the progression of glycolysis. This process involves the transportation of glutamate and glucose into the cytoplasm, followed by their participation in the tricarboxylic acid (TCA) cycle within the mitochondria. Conversely, heightened ROS levels hinder NFAT and c‐myc expression, resulting in glycolysis suppression. ROS can also induce the expression of other transcription factors in T cells, such as AP1 and NF‐κB, subsequently prompting cytokines release. TCR. T cell receptor; GSH, glutathione; GPX, glutathione peroxidase; ASCT2, alanine‐serine‐cysteine transporter; mTOR, mammalian target of rapamycin; NFAT, nuclear factor of activated T cells; AP‐1, activator protein‐1; IL, Interleukin.

T cells play essential roles in anticancer immune responses. However, TME creates an immunosuppressive environment, resulting in the suppression of cytotoxic T lymphocytes and thus leading to cancer progression. ROS has been studied as one crucial factor in the activation and metabolism of T cell.^184^, ^185^ Enhanced activity of metabolism in activated T cells results in ROS generation. The inappropriate elimination of ROS can lead to DNA damage and cell death. T cell receptor (TCR) signaling pathways are affected by ROS, activating several proximal and distal signaling pathways in T cells and impacting the activation of T cells.^184^ It has been shown that TCR stimulation induced the generation of ROS, leading to the activation of TFs such as a nuclear factor of activated T cells (NFAT), activator protein‐1 (AP‐1).^186^ Overexpression of ROS further inhibits the activity of mTOR and expression of NFAT and c‐Myc TFs, regulating metabolic reprogramming and T‐cell activation^187^, ^188^ (Figure 3). It has been demonstrated that activated neutrophils inhibited DNA synthesis in human T cells by increasing the superoxide levels. Further research suggested that the inhibition of DNA synthesis was associated with alterations in TCR signaling.^182^ Another study found that the viability of CD4 T cells decreased when cocultured with autologous granulocytes, and adding catalase could reverse this adverse effect.^189^ Therefore, all those studies indicate that ROS has a negative impact on T‐cell signaling, activation, proliferation, and viability.

Stimulating activated T cells, known as T‐cell blasts, leads to activation‐induced cell death (AICD), which is also accompanied by the release of ROS.^190^ As the significant ROS‐producing enzyme of phagocytes, NOX2 has been shown to be expressed in T‐cell blasts with the TCR stimulation, inducing the Fas‐dependent oxidation of ROS‐sensitive dye 2′,7′‐dichlorofluorescein diacetate (DCFDA) by NOX2‐derived ROS. This stimulation also induces transient oxidation that is not dependent on NOX‐2, indicating that two or more sources of ROS are involved in AICD.^191^ TCR‐triggered DCFDA oxidation in human CD4+ T cell blasts is associated with DUOX‐1.^192^ It can be concluded that TCR or Fas signaling in T‐cell blasts can produce ROS through NOX‐A, DUOX‐1, and perhaps other sources. Then ROS can activate the downstream signaling events, potentially responding to ERK signaling and triggering the expression of FasL. Fas ligation further activates NOX‐2, assisting the execution of the apoptotic program, AICD.^193^

It has been studied that increased levels of ROS or oxidative stress caused immunosuppression inside the TME via Tregs. Tregs are a subset of CD4^+^ T cells capable of suppressing immune responses and maintaining immune homeostasis and tolerance.^194^ A study found that Tregs derived from neutrophil cytosolic factor 1‐deficient mice with a lower level of ROS were less functional compared with those from the wild type. And this process can be blocked by thiol‐bearing antioxidants or NADPH oxidase inhibitors by decreasing the level of ROS.^195^ Increased ROS levels contributed to the hyperfunction of Tregs in psoriatic dermatitis.^196^ Metformin (complex I inhibitor) decreased the number of tumor infiltrating Tregs by preventing the differentiation of CD4 T cells into Tregs through Foxp3, the transcriptional regulator for metabolic reprogramming.^197^ Mitochondrial complex III is also crucial for inhibiting Treg function.^198^ SENP3 is a regulator of Treg stability and function, which can elucidate the underlying mechanism of the impact of ROS in Tregs‐mediated immunosuppression. SENP3 deficiency results in abnormal Tregs homeostasis and enhanced antitumor immunity. ROS can stabilize SENP3 quickly and thus maintain the function of Tregs via BACH2 deSUMOylation.^199^, ^200^

### 7.2 Myeloid‐derived suppressor cells

MDSCs are a subset of immunosuppressive immature myeloid cells that exist in various tumor patients.^201^ MDSCs can interact with other immune cells, such as T cells, to develop immunosuppression and anti‐inflammation, leading to the immune escape of tumors.^202^ In normal conditions, the number of MDSCs participating in the immune response is low. While in the early and advanced stages of cancer, the amount of MDSCs increases rapidly with the production of ROS.^203^ The sources of ROS in MDSCs mostly come from NOX2.^204^ ROS has been proven to play a crucial role in maintaining the undifferentiated state of MDSCs. A study showed that catalase treatment could induce myeloid cells to turn into macrophages by eliminating H_2_O_2_.^205^ Corzo et al.^204^ also found that the level of ROS produced in MDSCs increased in mice bearing five different tumors because of the upregulated NOX2 activity.

MDSCs could suppress T cell proliferation and response by producing ROS in mice and humans.^206^, ^207^ High levels of ROS and peroxynitrite have been detected in MDSCs in tumor, modifying T cell receptors and CD8 molecules. Further, CD8^+^ T cell would lose their ability to bind phosphorylated major histocompatibility complex and induce antigen‐specific tolerance of peripheral CD8^+^ T cell.^208^ Moreover, it has been suggested that MDSCs suppressed B cell proliferation and the production of antibodies to affect B cell‐mediated immune responses through NO and ROS.^209^

### 7.3 Macrophages

Macrophages consist of two polarized types, the proinflammatory M1‐like macrophages and the immunosuppressive M2‐like macrophages.^210^ Macrophages are the central immune cell in the TME, which can account for more than 50% of tumor mass in breast cancer.^211^ In the TME, TAMs play important roles in cancer cell growth, metastasis, and cancer cell evasion of the immune system.^212^ TAMs can transform their phenotypes and are mostly skewed towards the immunosuppressive type, M2‐like macrophages.^213^ CD163, the M2 macrophage surface marker, is associated with poor survival, metastasis, and grade in tumor patients.^214^, ^215^

Extracellular ROS is a positive factor in promoting the recruitment of monocyte and its differentiation into macrophages. Many studies suggested that H_2_O_2_ is a chemoattractant for monocytes.^216^, ^217^ Increased expression of uncoupling protein‐2 significantly decreases oxidative stress and intracellular ROS level in THP 1 human monocytes, preventing the migration and adhesion of monocytes and causing the reduction of macrophages. Besides, the ROS level is linked with the polarization of macrophages, which can transform the TME to be immunosuppressive.^218^, ^219^ Recent studies showed that the functions of ROS were different in the two types of macrophages. ROS and NO derived from inducible NO synthase are essential to the antimicrobial activity of M1 macrophages. Extracellular ROS is the critical factor in M2 macrophage differentiation. Elevated levels of ROS in the TME can contribute to the monocyte differentiation into M2 macrophages.^220^ Research in 2020 indicated that ROS was necessary for the polarization and function of M2 macrophages. The highly oxidized TME can maintain an immunosuppressive environment by promoting polarized TAM to M2 phenotype. And clinical redox‐active drugs can selectively target M2 macrophages, inhibiting their protumorigenesis and immunosuppression ability.^221^

### 7.4 Dendritic cells

Dendritic cells (DCs) are antigen‐presenting cells that are crucial to both innate and humoral immunity.^222^ Immature DCs can recognize and capture antigens through pattern recognition receptors expressed on the membrane, further activating itself into a mature state and migrating to the T cell zone in lymph nodes, where they present antigens to naïve T cells and activate cytolytic T cells by cross‐presentation.^223^ The internal and external environment influences the maturation of DCs and their ability to initiate immune responses, especially ROS level.^224^ Though ROS generally influences DCs function by affecting cytokine production, maturation, migration, and antigen presentation, the specific role of ROS derived from DCs in tumors remains unclear.^225^ NOX2 expressed in DCs could persistently produce low levels of ROS and promote cross‐presentation with the regulation of phagosomal and endosomal pH, which further could be blocked in NOX2‐defective DCs and DCs treated with NOX2 inhibitor.^226^ It is also indicated that the ROS in DCs regulated the immune response against cancer. The production of ROS increased in several subtypes of DCs in the process of cross‐presentation to cytotoxic CD8^+^ T cells. NOX2 is recruited to the phagosome, leading to alkalization of it by low‐level production of ROS. And the alkalization of phagosome can result in structural preservation of the internalized antigen presented by MHC I complex. Therefore, ROS produced by DCs can affect the immune response of CD8^+^ T cells and tumoral antigens.^227^, ^228^ High levels of ROS in the TME can suppress the function of DCs. ROS can enter DCs by several pathways, including diffusion across the plasma membrane or extracellular vesicles released by tumor cells.^225^ Chougnet et al.^229^ suggested that ROS derived from dysfunctional mitochondria have an inhibitory effect on the cross‐presentation ability of DCs. Besides, DCs maturation would be repressed by endoplasmic reticulum stress caused by ROS.^230^

### 7.5 Natural killer cells

Natural killer (NK) cells are lymphocytes that can directly eliminate infected or tumor cells without specific antigens.^231^ The proliferation of NK cells is associated with the prognosis of many solid cancers, and the cytolytic activity of NK cells correlates with tumor progression.^232^ In TME, ROS is regarded as having a detrimental role in the viability and function of NK cells. NK cells are susceptible to apoptosis induced by oxidative stress. And ROS in NK cells derived from tryptophane catabolite kynurenine or lactate could promote their apoptosis.^233^ In non‐small‐cell lung cancer, activation of antioxidative pathways such as thioredoxin increases the ability of NK cells to resist oxidative stress and possess a higher thiol capacity.^234^ The high level of ROS in the TME is considered one of the reasons leading to the exhaustion of NK cells. Moreover, Aydin et al.^235^ demonstrated that ROS derived from NOX medullary cells might suppress the release of IFN‐y from NK cells, thus promoting pulmonary metastasis of melanoma in mice. And by fostering antioxidant capacity or decreasing ROS levels, NK cells could maintain their normal function and avoid exhaustion.^236^

### 7.6 Neutrophils

Neutrophils are crucial to the innate immune system. Neutrophils are identified as having significant cellular plasticity and a wide range of subtypes, leading to the functional heterogeneity of both pro and antitumorigenesis functions of neutrophils in TME.^237^, ^238^ Many studies have indicated that neutrophils exerted cytotoxic effects regulated by ROS in TME. A vivo study suggested that H_2_O_2_ derived from neutrophils could kill metastatic breast cancer cells in the pre‐metastatic lung, thus reducing the distant growth of primary tumors. And when used the H_2_O_2_ scavenger catalase, this effect would be abolished, indicating that it was H_2_O_2_ that was responsible for tumor cell apoptosis.^239^ Another study focused on the underlying mechanism and found that H_2_O_2_ induced apoptosis of cancer cells via the TRPM2 ion channel.^240^ Besides those direct impacts, neutrophil ROS also regulates the functions of other immune cells in the TME. H_2_O_2_ derived from neutrophils can inhibit the function of NK cells, decreasing tumor clearance and promoting lung colonization in mice with metastatic breast cancer.^241^ Neutrophils‐derived ROS can also impair the proliferation of T cells and influence the production of the protumorigenic cytokine IL‐17.^242^ Recently, several studies have indicated that neutrophil ROS correlated with tumor initiation. Another study observed that colon tumors were more invasive in mice with specific deletion of GPX4, a ROS scavenger, after repeatedly injecting the carcinogenic agent azoxymethane. GPX4‐deficient myeloid cells could produce excessive ROS, increasing the mutational burden of colonic epithelial cells and initiating more aggressive tumors.^243^ A study in 2020 found that ROS derived from neutrophils could enhance the DNA damage that helps the early tumor development in primary lung cancer. Neutrophils contribute directly to neoplastic transformation by amplifying the genotoxicity of urethane in lung cells via ROS.^244^

NETosis (neutrophil cell death with releasing neutrophil extracellular traps) has been found to regulate tumor progression. Depletion of albumin and free thiols can cause a systemic redox imbalance, leading to ROS accumulation in neutrophils and further triggering NETosis in the lung, promoting lung metastasis in vivo.^245^

### 7.7 B cells

It has been suggested that B cells receptor (BCR) activated by antigen stimulation can induce elevated levels of ROS.^246^ Hematopoietic stem cells differentiate into multipotent progenitor cells and common lymphoid progenitor cells in the bone marrow and further transform into mature B cells.^247^ Mature B cells can differentiate into antibody‐secreting plasma B cells, memory B cells, and cytokine‐producing B cells when combined with antigens or other molecules.^248^ ROS is an essential modulator in the maturation, activation, and proliferation of B cells, also involved in the apoptosis of B cells by mediating oxidative stress.^249^ ROS is proven to affect some physiological processes of B‐lineage lymphoid cells through the signaling pathway of the tyrosine phosphorylation of proteins.^250^ In the tumor immune responses, B cells are involved in the clonal expansion of tumor antigen‐specific T cells via antigen presentation and antibody secretion, suppressing the brain tumor and modulating B cell HLA genes as an antitumor vaccine.^251^, ^252^ Elevated ROS levels were found in several B‐cell malignancies, such as B‐cell acute lymphoblastic leukemia, chronic lymphocytic leukemia (CLL), and B‐cell lymphomas.^253^, ^254^ Moreover, B‐cell‐derived cancer cells are sensitive to prooxidant treatments, which may be related to the specific metabolic reprogramming of these cells.^255^

## 8 ROS AND CANCER IMMUNOTHERAPY

### 8.1 Synergistic effects of ROS and immunotherapy

**FIGURE 4:**
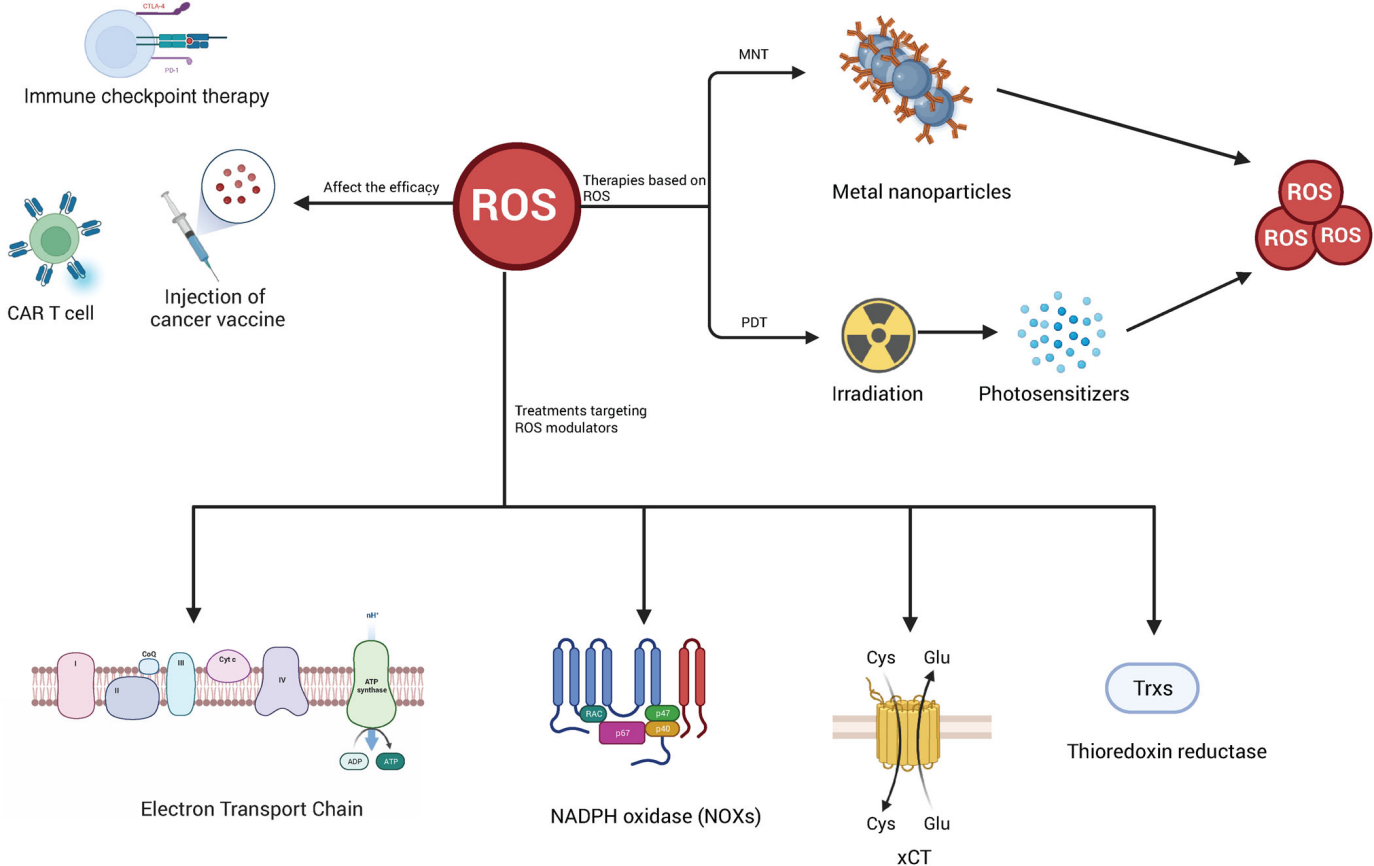
The association between ROS and cancer immunotherapy. The role of ROS is closely intertwined with cancer immunotherapy. ROS profoundly influences the efficacy of various immunotherapies, including immune checkpoint therapy, CAR‐T cells, and cancer vaccines. Notably, novel immunotherapies such as photodynamic therapy (PDT) and metal nanoparticle therapy (MNT) have been developed based on ROS. Targeting ROS modulators shows promising potential for achieving significant clinical effects. xCT, the glutamate/cystine antiporter solute carrier family 7 member 11 (SLC7A11).

As highlighted earlier, the levels of ROS intricately intertwine with the immune response, thereby significantly impacting the efficacy of cancer immunotherapy. Notably, ROS plays critical roles in diverse forms of immunotherapy, encompassing immune checkpoint blockade, chimeric antigen receptors (CARs), and cancer vaccines. In the subsequent section, we will elucidate the intricate relationship between ROS and these distinct modalities of immunotherapy (Figure 4).

#### 8.1.1 Immune checkpoint blockade

The successful targeting of immune checkpoints, such as CTLA‐4 and PD1/PDL1, represents a significant advancement in cancer therapy over the past decade.^256^ Studies have proved that ROS plays essential role in the response of ICB. In ovarian cancer, a novel synthesis stimulating ROS production has exhibited synergistic effects with anti‐CTLA‐4 therapy, effectively impeding tumor metastasis progression and promoting the infiltration of T cells.^257^ Furthermore, heightened levels of ROS and oxidative stress within the TME have been associated with intensified immunosuppression mediated by Tregs. Apoptotic Tregs have demonstrated greater efficacy in suppressing T cell activation and have been implicated in undermining the therapeutic efficacy of PD‐L1 blockade in mouse models bearing tumors.^183^ Additionally, combined treatment involving an anti‐PD‐L1 agent and the photosensitizer (PS) indocyanine green (ICG) has been observed to induce ROS during photodynamic therapy (PDT), resulting in enhanced infiltration of CD8^+^ T cells within tumors and a concomitant attenuation in both primary and metastatic tumor growth.^258^ Notably, a separate study has proposed that the application of a ROS generator can heighten the effectiveness of PD‐1 blockade, even leading to the complete recovery of certain mice in an immunogenic colorectal cancer xenograft model.^259^ Moreover, the modulation of ROS generation through Nano‐FdUMP has been demonstrated to enhance the efficacy of immunogenic cell death induced by Nano‐Folox, with subsequent combined treatments involving Nano‐FdUMP/Nano‐Folox and anti‐PD‐L1 antibodies displaying the capacity to suppress colorectal cancer liver metastasis and enhance overall survival in murine models.^260^

#### 8.1.2 Chimeric antigen receptors

Therapies involving engineered T cells, notably CARs (CAR‐T cells), confer the dual advantage of antigen recognition and T cell response, showcasing clinical efficacy in both solid tumors and hematological malignancies.^261^, ^262^ Specifically, B7‐H3 CAR‐T cells have demonstrated effective inhibition of non‐small cell lung cancer, concurrently modulating tumor glucose metabolism through the activation of the ROS pathway.^263^ In the context of CLL, tumor cells can disrupt T cell mitochondria, enhancing mitochondrial respiration, membrane potential, and the production of ROS, consequently diminishing the effectiveness of CAR‐T cell therapies.^264^ Yoo et al.^265^ found that application of prodrugs of ROS accelerators increased accumulation of ROS specifically within the TME, significantly augmenting the cytotoxicity of CAR‐T cells in Burkitt lymphoma cell lines Raji and Daudi. Furthermore, in a mouse model of myeloma, c‐reactive protein has been found to induce increased levels of ROS and oxidized GSH in CD8^+^ T cells, consequently suppressing the immune response of CAR‐T treatments.^266^ It is also worth mentioning that mesenchymal stem cells have the capacity to modulate ROS levels, thereby impeding the formation of the NLRP3 inflammasome and ultimately hindering the efficacy of CAR‐T therapies.^267^

#### 8.1.3 Cancer vaccine

Cancer vaccine utilizes tumor‐associated antigen and tumor‐specific antigen to initiate the immune system of cancer patients, effectively triggering tumor antigen‐specific cellular immune responses that target and eliminate cancer cells.^268^ Research has highlighted the crucial role of ROS in the antitumor activity induced by cationic liposome‐based cancer vaccines.^269^ For instance, the development of a folic acid‐modified liposome loaded with chlorin e6 (FA‐Lipo‐Ce6) as a DC vaccine has proven to be a promising approach. Upon irradiation, the loaded chlorin e6 can be activated, leading to the generation of ROS at the tumor site, subsequently inducing cancer cell apoptosis and the exposure of tumor‐associated antigens.^270^ In a similar vein, Zhang et al.^271^ demonstrated the efficacy of a programmable nanomedicine based on supramolecular assembly. This nanomedicine, upon accumulation at the tumor site, triggered increased ROS levels within the TME, thereby facilitating the dissociation of the programmable immune activation nanomedicine and the subsequent release of components that guide the killing of cancer cells and the release of antigens.^271^ Additionally, Xu et al.^272^ observed that a personalized cancer vaccine in the form of reduced graphene oxide nanosheets (RGO‐PEG) could enhance the production of ROS in DCs, thereby facilitating the processing and presentation of antigens to T cells for the specific elimination of cancer cells.

### 8.2 Photodynamic immunotherapy based on ROS

PDT is a noninvasive treatment that harnesses the power of ROS generated by PSs to target and combat tumors.^273^ PSs are compounds capable of absorbing specific wavelengths of ultraviolet light, resulting in the production of free radicals or ions and initiating photopolymerization. Upon irradiation, PSs activate and transfer energy to oxygen, leading to the generation of ROS. PDT is known for its reduced adverse effects and systemic toxicity in comparison with conventional radiotherapy and chemotherapy.^274^ By inducing oxidative stress, PDT stimulates the activation of innate immune cells and the expression of IL‐1, IL‐6, and TNF‐υ. Notably, studies have shown that fluorine‐assembled photodynamic immunotherapy (PMPt) is able to generate significant levels of ROS upon laser irradiation, releasing cisplatin‐conjugated PMPt and inhibiting tumor growth by targeting Treg cells and MDSCs.^275^ Several preclinical investigations have demonstrated that combining immune checkpoint inhibitors with PDT can lead to enhanced antitumor effects.^276^ For instance, in the CT26 bilateral tumor model, the administration of PD‐L1 blockade alongside light‐induced ROS production led to increased infiltration of CD8^+^ T cells in both primary and distal tumors, ultimately suppressing tumor progression.^277^ However, the efficacy of PDTs in clinical settings is often hindered by insufficient oxygen supply and hypoxia within the TME of solid tumors, resulting in reduced ROS production from PSs. Consequently, various research efforts have focused on strategies to generate ROS in situ or deliver oxygen to the TME, aiming to improve the effectiveness of PDTs.^278^, ^279^ Recent studies have introduced innovative approaches such as a catalytic nano platform (nGO‐hemin‐Ce6),^280^ and piezo‐photocatalytic therapy,^281^ both of which have exhibited promising tumor‐suppression effects through the induction of intracellular ROS and subsequent apoptosis of cancer cells. Furthermore, the utilization of cancer cell membrane‐camouflaged gold nanocages loaded with doxorubicin (DOX) and I‐buthionine sulfoximine (BSO) has been found to evoke ferroptosis through the consumption of GSH and ROS generation. This approach, combined with photothermal therapy, has demonstrated the potential to repolarize TAMs from a protumor M2 phenotype to an antitumor M1 phenotype, thus improving the immune response against tumors.^282^

### 8.3 Metallic immunotherapy based on ROS

Metal nanoparticles (MNPs), characterized by their small size and high tensile strength, have demonstrated the ability to penetrate solid tumors alongside target materials, making them a promising tool in cancer therapy.^283^ MNPs exert their therapeutic effects by generating ROS through interactions with cell mitochondria. MNPs such as iron (Fe), copper (Cu), and manganese (Mn) can impede electron transfer and influence the mitochondrial membrane potential, resulting in the accumulation of excessive ROS via Fenton and Fenton‐like reactions.^284^, ^285^, ^286^ For instance, iron oxide has been shown to convert intracellular H_2_O_2_ into reactive hydroxyl radicals, enhancing oxidative stress and inducing cancer cell death through heterogeneous Fenton reactions.^287^ Elevated levels of ROS in the TME and the systemic circulation have been found to promote the release of proinflammatory cytokines, thereby enhancing the immune response. Moreover, studies have indicated that magnetite IONPs can drive the polarization of macrophages, shifting M2 macrophages to an M1 phenotype by downregulating the expression of M2‐associated arginase 1.^288^ Additionally, in vivo experiments with tumor models treated with gold nanospheres containing the pardaxin (FAL) peptide and ICG demonstrated increased ROS levels compared with the untreated group, suggesting a synergistic antitumor effect resulting from the combination of PDT and photothermal therapy (PTT).^289^ By enhancing the structure and composition of metal‐organic frameworks, researchers aim to develop a new clinically applicable nanotechnology platform that facilitates immune stimulation within the TME, enabling catalytic PDT and immunogenic cell death, thus improving cancer immunotherapy.^290^ Notably, Xiang et al.^291^ have developed a novel polyphenol‐metal nanocarrier for the fabrication of core–shell nanoparticles containing cisplatin, which remain stable in circulation and specifically disassemble at the tumor site due to polyphenol dissociation induced by ROS. Furthermore, SnFe_2_O_4_ nanocrystals have shown promise as heterogeneous Fenton catalysts and can be delivered to the lungs through respiratory movement, exhibiting antitumor effects via the enhanced permeability and retention effect or magnetically guided drug targeting technology.^292^

### 8.4 Treatments targeting ROS modulators in cancer as a new immunotherapy

Targeting ROS modulators has been a novel immunotherapy. Among those modulators, we focused on the promising and widely studied component, including mitochondrial ETC, NOXs, xCT, and thioredoxin reductases (TrxRs). Drugs targeting these ROS modulators have been developed, even undergoing clinical trials (Table 2).

**TABLE 2:** The mechanisms and research status of typical drugs that target ROS modulators mentioned above.

Agent	Mechanisms	Status	Clinical trial no.	References
Targeting mitochondrial electron transport chain complexes (ETC)				
Elesclomol	Transport copper ions into cells and cause the oligomerization of lipoylated dihydrolipoamide S‐acetyl‐transferase	Clinical trials phase I/II/III	NCT00522834^a^	^335^, ^336^
MitoQ	Inactivate mitochondrial tumor necrosis factor, target mitochondrial ETC I, III, and IV	Clinical trials phase I/II	NCT06069245^a^	^337^, ^338^, ^339^
DT‐010	Inhibit mitochondrial respiratory chain complex II and the glycolysis pathway, increase DOX accumulation in cells	Preclinical study	–	^340^, ^341^
Targeting NOXs				
GKT137831	Inhibit NOX1, NOX4, and NOX5, downregulate MAPK pathway	Clinical trials phase I/II	NCT03226067^a^	^342^, ^343^, ^344^, ^345^
NOS31	Inhibit NOX1, NOX4	Preclinical study	–	^311^
Capsaicin	Inhibit SIRT1/NOX4 signaling pathway, inhibit NAD+‐dependent SIRT1 deacetylase, suppress activated microglia‐derived NADPH oxidase	Clinical trials phase I/II/III	NCT00993070^a^	^346^, ^347^, ^348^, ^349^
Targeting xCT				
Erastin	INHIBIT VDAC2/VDAC3, block GSH synthesis, increase lipid peroxidation and lipid ROS	Preclinical study	–	^350^, ^351^
Sulfasalazine	Regulate CD44v9‐xCT system, inhibit GPX4, trigger ferroptosis	FDA approved	–	^352^, ^353^
Aspirin	Inhibit xCT, induce ROS‐dependent depolarization and activate voltage‐gated Ca2+ entry	FDA approved	–	^354^, ^355^
Targeting TrxRs				
Auranofin	Reduce activity of TrxR1, decrease GSH levels, increase intracellular ROS levels, inhibit the activity of STAT3 and telomerase	FDA approved	–	^330^, ^356^, ^357^
Ethaselen	Inhibit TrxR1 and NF‐κB, increase ratio of Bax to B cell lympoma‐2 protein	Preclinical study	–	^333^, ^358^
Ruthenium(II) complex	Induce the potential loss of mitochondria membrane, inhibit TrxR activity, promote oxidative stress‐sensitive MAPK	Preclinical study	–	^359^, ^360^, ^361^

Abbreviations: DOX, doxorubicin; GSH, glutathione; MAPK, mitogen activated protein kinase; NOXs, nicotinamide adenine dinucleotide phosphate oxidases; NADPH, nicotinamide adenine dinucleotide phosphate; SIRT1, sirtuin1; STAT3, signal transducer and activator of transcription 3; TrxRs, thioredoxin reductases; VDAC, voltage‐dependent anion channel; xCT, the glutamate/cystine antiporter solute carrier family 7 member 11 (SLC7A11); clinical trials are available from: http://ClinicalTrials.gov/.

^a^ Trials with reported results.

#### 8.4.1 Targeting mitochondrial metabolism

The cores of solid tumors are mostly with poor vascularization and low oxygen level. And these tumors could maintain the function of respiration since the ETC could generally work at low oxygen levels.^293^, ^294^ Moreover, diminishing ETC function can suppress the TCA cycle,^295^ further increasing the levels of ROS and suppressing tumor growth. A study conducted in several models of liver cancer, including cell line, organoid, and murine xenografts, found that inhibition of ETC complex I and III could suppress cancer cell growth and clonogenicity by regulating the generation of ROS, inducing apoptosis and reducing ATP production.^296^ Therefore, the mitochondria ETC can be a potential therapeutic target in tumors. Elesclomol is a chemotherapeutic drug treating metastatic melanoma.^297^ It can transport copper ions into the cancer cell, which can be selectively enriched in mitochondria and affect the lipoylated TCA cycle proteins. Finally elesclomol results in a novel form of cell death termed cuproptosis.^298^, ^299^ MitoQ is an antioxidant targeting mitochondria that can localize to mitochondrial membrane and exert a long‐lasting function.^300^ It can suppress the initiation of hepatocarcinoma by regulating mtROS, connexins and the expression of p53 as a chemopreventive treatment.^301^ DT‐010, a synthetic product of danshensu and tetramethylpyrazine that can kill breast cancer cells via inhibiting ETC complex II, which can further be reversed by NAC, a cysteine compound that has an antioxidant effect.^302^

#### 8.4.2 Targeting NOXs

NOXs can produce H_2_O_2_ and O_2_^−^, one kind of essential molecules that regulate cellular redox levels, growth‐related responses, and cancer progression.^303^, ^304^ Growing evidence has suggested that ROS derived from NOXs family may contribute to genomic instability, promoting the initiation of cancer and drug resistance.^305^, ^306^ A study conducted in vivo and in vitro found that NOX5‐derived ROS could mediate epithelial cell proliferation and survival in prostate cancer.^307^ The level of NOX2 increased in gastric cancer and promoted angiogenesis and tumorigenesis, thus rendering NOX2 a potential therapeutic target.^308^ GKT137831 is a selective NOX4 inhibitor that can attenuate the generation of hypoxia‐induced H_2_O_2_ in human pulmonary artery smooth muscle cells and human pulmonary artery endothelial cells.^309^ An in vitro and in vivo study suggested that GKT137831 could inhibit glucose and glutamine metabolic phenotypes and cancer progression when combined with 2‐DG.^310^ NOS31 is a novel specific NOX1 inhibitor identified by Yamamoto et al. in 2017, which can specifically suppress the production of NOX1‐derived ROS. Further study found that NOS31 could inhibit the proliferation of several colon and stomach cancer cells overexpressing NOX1, while those cells that expressed low levels of NOX1 would not be influenced.^311^ Capsaicin, a natural component extracted from chili peppers, is regarded as a potential NOX inhibitor. Capsaicin suppresses the activity of NOXs and induces an abnormal ROS stress to selectively kill k‐ras‐transformed cells, which occurs in >90% of pancreatic ductal carcinoma.^312^

#### 8.4.3 Targeting xCT

xCT, known as the glutamate/cystine antiporter solute carrier family 7 member 11 (SLC7A11), is a molecule of the antiporter system x_c_^−^ that regulates the exchange of extracellular l‐cystine and intracellular l‐glutamate across the plasma membrane for GSH synthesis. xCT is involved in modulating the survival of somatic and immune cells and the progression of cancer cells, further promoting chemotherapy resistance.^313^ It is indicated that the elevated expression level of xCT is associated with the recurrence of colorectal cancer and esophageal squamous cell carcinoma.^314^, ^315^ Moreover, the low expression level of xCT leads to a decreased level of GSH and an increased level of ROS, further causing tumor cell death and thus indicating xCT as a potential therapeutic target.^316^

Sulfasalazine is an xCT inhibitor with chemosensitizing efficacy.^317^ Sulfasalazine inhibits xCT, impairing the ROS defense system and promoting the efficacy of cisplatin and DOX in CD133‐positive HCC.^318^ Sulfasalazine can also downregulate the levels of GSH, increasing the effectiveness of celastrol in celastrol‐resistant glioma cells characterized by overexpression of xCT.^319^ Erastin is a voltage‐dependent, anion channel‐binding xCT inhibitor. Erastin can induce an iron‐dependent cell death, known as ferroptosis.^16^ When combined with cannabidiol, Erastin exhibits a synergistic increase in ROS levels and suppresses glioma stem cell viability, invasion, and self‐renewal.^320^ In addition, aspirin can potently inhibit xCT and decrease GSH, inducing ROS accumulation in head and neck cancer cells when combined with sorafenib.^321^

#### 8.4.4 Targeting thioredoxin reductases

TrxRs are a group of selenium‐containing pyridine nucleotide‐disulfide oxidoreductases.^322^ TrxRs can catalyze the NADPH‐dependent reduction of the redox protein Trx to regulate the intracellular oxidative balance.^323^ The thioredoxin system affects many biological functions, such as resisting oxidative stress, deoxyribonucleotide synthesis, and cell apoptosis.^324^, ^325^ Several studies have suggested that upregulating the expression of TrxR‐induced drug‐specific cytotoxic responses while knocking down TrxR could improve the efficacy of several chemotherapeutics.^326^, ^327^ Therefore, TrxR is regarded as a potential therapeutic target for some chemotherapy‐resistant cancers. Auranofin, used to treat RA, has been found the ability to induce cancer cell death.^328^ In leukemic cells that overexpressed TrxR, auranofin is more productive than DOX in suppressing cancer cell proliferation and inducing apoptosis.^329^ It was also reported that in vitro study, auranofin could decrease cancer side population cells and downregulate stem cell markers to prevent colony formation. Moreover, in vivo, auranofin could suppress the initiation of tumors.^330^ Ethaselen is another compound that can inhibit TrxR1 and exhibit a synergistic effect when combined with certain chemotherapeutic drugs.^331^, ^332^ Ethaselen could increase the efficacy of cisplatin in cisplatin‐resistant human erythrocyte leukemic K562/CDDP cells. Besides, it could also potently increase levels of ROS by inhibiting NF‐kB, leading to the release of cytochrome release and cancer cell apoptosis.^333^ Zeng et al.^334^ synthesized Ruthenium(II) complex 4, a more efficacious compound than cisplatin in inhibiting MDR A549R cell proliferation. It can inhibit the expression of TrxR and cause the accumulation of intracellular ROS, resulting in mitochondrial dysfunction and an arrested cell cycle that induce apoptosis of A549R cells.^334^

## 9 CONCLUSIONS AND PROSPECTS

As one of the molecular reactive species, ROS plays a vital role in cell signaling and various biochemical reactions. ROS can regulate epigenetic modifications to exhibit a long‐term impact on cells. Subsequent oxidative stress by ROS is proved to be directly associated with multiple types of cell death, including apoptosis, necrosis, and autophagy. Moreover, transcriptional factors are connected with redox regulation for maintaining the cell physiology. While in abnormal pathological conditions, ROS is still inevitable to be involved in the process of inflammation. The interaction between ROS and inflammation is a feedback loop, where inflammation state result in redox imbalances and redox status regulates the inflammation response. Based on this relation, the functions of ROS in inflammation‐induced tumorigenesis are further elucidated. Many research have emphasized the crucial roles of ROS and inflammation in tumorigenesis. Notably, ROS is found to enhance DNA damage and genomic instability caused by inflammation, accelerating neoplasm. After the development of tumor, ROS still exhibit its unique functions in cancer. ROS is related to the proliferation, metabolism, invasion and metastasis of cancer cells. The functions of ROS in cancer are intricate and multifaceted. Typically, cancer cells exhibit a higher level of ROS accumulation. When ROS levels surpass the cellular antioxidant capacity, excessive oxidative stress will induce genomic instability and subsequent cell death in cancer cells. In those processes, NRF2 is crucial in both tumor progression and redox homeostasis. Loss of NRF2 triggers tumorigenesis, while abnormal activation and accumulation of NRF2 promote tumor progression. Besides, other antioxidants also contribute to the survival of cancer cells. Therefore, supplementation of exogenous antioxidants, including NAC and vitamin E, is regarded as a therapy for interfering with tumor process. More specifically, ROS can influence cancer immunity in the TME, further exerting its function on tumors. Current studies have shown that ROS is associated with almost all kinds of immune cells in the TME, including T cells, MDSCs, macrophages, DCs, NK cells, neutrophils, and B cells. A clear understanding of the relationship between ROS and immune cells will help further recognize the immune microenvironment of tumors and develop new therapeutic approaches. Based on its impact on cancer immunity, ROS is further found to play a vital role in cancer immunotherapy. However, the exact functions of ROS in immunotherapy are still needed to be explored. In some cases, accumulation of ROS can enhance the immune response of ICB or CAR‐T, while in other cases, increased levels of ROS impair the efficacy of immunotherapy. Despite those widely applied treatment, two novel types of immunotherapies directly based on ROS, PDT and MNPs, is promising to achieve clinical benefits. Meanwhile, the regulatory molecules in the process of ROS generation, such as the mitochondrial transport chain, NOXs, and xCT, are also worthy of attention. Currently, many basic studies have proved that drugs targeting ROS modulators have a certain effect on inhibiting the growth of tumor cells, but unfortunately, very few can be used in clinical practice.

Certainly, the complex interplay between ROS, inflammation, and cancer presents several key areas that warrant focused investigation and the development of targeted therapeutic strategies. Moving forward, a more comprehensive understanding of the precise molecular mechanisms through which ROS modulate inflammatory pathways and impact cancer progression is crucial. Unraveling the specific signaling cascades and crosstalk between ROS and proinflammatory TFs could provide insights into their regulatory roles within the TME. Moreover, exploring the intricate connections between ROS and the immune response, particularly their influence on immune cell polarization and modulation of checkpoint molecules, is essential for designing effective immunotherapies. Investigating the implications of ROS on the tumor mutational burden, neoantigen presentation, and the efficacy of cancer vaccines can guide the development of personalized immunotherapeutic interventions targeting ROS‐associated pathways. In parallel, the development of innovative ROS‐targeted therapies for cancer treatment holds promise. The design of targeted ROS modulators, such as scavengers or ROS‐generating agents, specific to the TME, could provide novel strategies for controlling tumor progression and preserving antitumor immune responses. Integrating advanced imaging techniques for real‐time ROS monitoring and nanoparticle‐based delivery systems for ROS modulators can further enhance the efficacy of ROS‐based therapies.

Additionally, understanding the diverse ROS profiles in different cancer types and their molecular subtypes can facilitate the development of tailored treatment approaches. Investigating the impact of ROS on tumor heterogeneity, metastatic potential, and therapeutic resistance is essential for devising effective strategies specific to different cancer subtypes. Integrating multi‐omics approaches and advanced computational models can accelerate the identification of ROS‐associated biomarkers and therapeutic targets, ultimately contributing to precision oncology.

In conclusion, future research efforts should focus on unraveling the intricate molecular networks involving ROS in inflammation and cancer. This requires interdisciplinary collaborations and the integration of cutting‐edge technologies to decipher complex signaling pathways and spatial dynamics within the TME. By advancing our understanding of ROS‐mediated mechanisms and their implications for cancer therapy, the future holds the potential for transformative advancements in precision oncology and the development of personalized ROS‐targeted treatments.

## AUTHOR CONTRIBUTIONS

*Study design and manuscript writing*: Yunfei Yu, Shengzhuo Liu, and Luchen Yang. *Language editing*: Pan Song and Xiaoyang Liu. *Figure and table making*: Zhenghuan Liu and Xin Yan. Qiang Dong was responsible for the whole work design and supervising the execution. All authors have read and approved the final manuscript.

## CONFLICT OF INTEREST STATEMENT

The authors declare that there is no conflict of interest for each author.

## FUNDING INFORMATION

Not applicable.

## ETHICS STATEMENT

Not applicable.

## Data Availability

Not applicable.
